# Emotion Recognition Based on Multichannel Physiological Signals with Comprehensive Nonlinear Processing

**DOI:** 10.3390/s18113886

**Published:** 2018-11-11

**Authors:** Xingxing Zhang, Chao Xu, Wanli Xue, Jing Hu, Yongchuan He, Mengxin Gao

**Affiliations:** 1 College of Intelligence and Computing, Tianjin University, Tianjin 300350, China; zhangxingxing@tju.edu.cn (X.Z.); xuchao@tju.edu.cn (C.X.); mavis_huhu@tju.edu.cn (J.H.); 2 School of Computer Science and Engineering, Tianjin University of Technology, Tianjin 300384, China; 3 Shenzhen Graduate School, Peking University, Shenzhen 518055, China; yongchuan_he@163.com; 4 Department of Economics, Pennsylvania State University, State College, PA 16803, USA; mbg5491@psu.edu

**Keywords:** emotion recognition, multichannel physiological signals, comprehensive nonlinear processing, KPCA and GBDT

## Abstract

Multichannel physiological datasets are usually nonlinear and separable in the field of emotion recognition. Many researchers have applied linear or partial nonlinear processing in feature reduction and classification, but these applications did not work well. Therefore, this paper proposed a comprehensive nonlinear method to solve this problem. On the one hand, as traditional feature reduction may cause the loss of significant amounts of feature information, Kernel Principal Component Analysis (KPCA) based on radial basis function (RBF) was introduced to map the data into a high-dimensional space, extract the nonlinear information of the features, and then reduce the dimension. This method can provide many features carrying information about the structure in the physiological dataset. On the other hand, considering its advantages of predictive power and feature selection from a large number of features, Gradient Boosting Decision Tree (GBDT) was used as a nonlinear ensemble classifier to improve the recognition accuracy. The comprehensive nonlinear processing method had a great performance on our physiological dataset. Classification accuracy of four emotions in 29 participants achieved 93.42%.

## 1. Introduction

The purpose of emotion recognition is to build a harmonious human-machine environment by giving a computer the ability to recognize human emotion. It has been widely applied in many fields, such as disease diagnosis [1,2], criminal investigation [3,4], and distance education [5,6]. At present, there are two commonly used methods for emotion recognition. One is to detect physiological signals [7,8,9,10,11,12,13]; the other is to detect emotion behaviors such as facial expression [14,15], speech [16] and gesture [17]. By contrast, physiological signals are more reliable, they are not controlled by participants’ subjective consciousness. Therefore, emotion recognition based on physiological signals has become a hot topic in the field of human-computer interaction.

Physiological signals are so easily influenced by many factors such as the human body and external environment, that they bring great difficulties to data acquisition. Besides, the feature extraction is blind due to the lack of prior knowledge about emotion recognition, which results in many features including lots of related, redundant, and nonlinear features. These two reasons make it difficult to distinguish the emotion categories from physiological signals, especially multichannel physiological signals, because there are interactions between the features from different signals. Many researchers have done a lot of work from feature reduction and classification to overcome this difficulty.

Most of the traditional emotion recognition models have adopted linear or partial nonlinear methods (such as Principal Component Analysis & Support Vector Machine (PCA & SVM) and PCA & K-Nearest Neighbor (PCA & KNN)) to solve this problem. However, these methods did not work well on the nonlinear physiological dataset and they did not consider the interactions between features. Therefore, it is necessary for emotion recognition from the nonlinear multichannel physiological dataset to undertake comprehensive nonlinear processing.

We consider that the comprehensive nonlinear processing includes both nonlinear feature reduction and nonlinear classification. For nonlinear feature reduction, considering the loss of feature information caused by traditional dimension reduction, we think it is an effective way to map the features into a high-dimensional space by a kernel function and then reduce dimensions. With the increase of dimensions, there may be more distinguishable physiological features [18]. Specifically, Kernel Principal Component Analysis (KPCA) based on radial basis function (RBF) kernel was adopted to handle this problem, with details in Section 3.1. The increase of dimension may also produce some redundant features that will have an impact on classification. For this problem, we used Gradient Boosting Decision Tree (GBDT) as a nonlinear classifier to improve the recognition accuracy, since GBDT is an ensemble model constructed by multiple decision trees, which can select and combine significant features for classification automatically [19].

**Figure 1:**
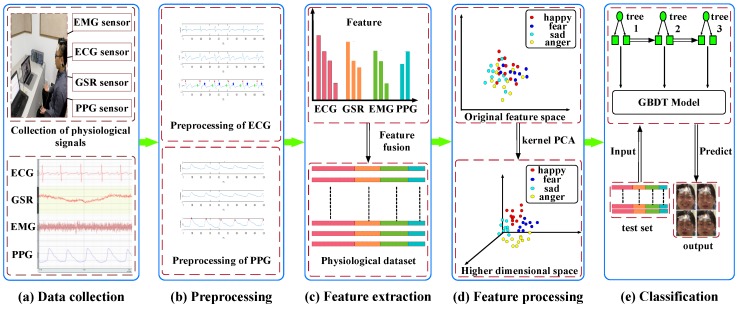
The process of emotion recognition framework.

Based on the above analysis, this paper proposed an emotion recognition framework using comprehensive nonlinear processing, as shown in Figure 1. Firstly, four physiological signals (electrocardiogram (ECG), galvanic skin response (GSR), electromyography (EMG) and photoplethysmography (PPG)) were collected under four induced emotions (pleasure, fear, sadness, and anger), then they were segmented to short-term signals to reduce the required length of signals. Secondly, the short-term signal segments were preprocessed, and the characteristic waveforms were detected from ECG and PPG signals. Thirdly, emotion-relevant features were extracted from the processed signals and all features were fused to construct the physiological dataset. Fourthly, KPCA was used to map the data into a high-dimensional space and then reduce feature dimension. Finally, GBDT model was estimated to predict the emotion of test set. The result analysis of the comprehensive nonlinear processing in Section 4.4 shows that the method could greatly assist the emotion recognition system towards multichannel physiological signals, and other systems related to the multichannel electrical signals.

Our contributions are:

- A comprehensive nonlinear processing method was proposed to improve the accuracy of emotion recognition from multichannel physiological signals.
- KPCA based on RBF kernel was adopted to solve the loss of feature information caused by dimension reduction in the physiological datasets (especially nonlinear multichannel datasets).
- GBDT was used to resolve the poor performance of general classifier under a nonlinear multichannel physiological dataset with many features from different signals.
- The classification accuracies of four physiological signals and four emotions were compared by different models.

The paper is organized as follows: Section 2 gives a brief overview of related work in the field of emotion recognition based on physiological signals. Section 3 presents the details of comprehensive nonlinear processing method. Section 4 describes the acquisition experiment of physiological signals and compares the classification results of different models. Section 5 concludes our work and outlines further work.

## 2. Related Work

As signal processing and sensor technology are advancing, we can continuously record a participant’s physiological signals if he wears bio-sensors. Increased attention has been paid to emotion recognition based on physiological signals. Different emotion induction plans are designed to collect various physiological signals, then many feature processing and classification methods are used for emotion recognition from these physiological signals.

### 2.1. Multiple Physiological Signals

Acquisition of a high-quality database of physiological signals is the first step for emotion recognition. In the field of emotion recognition towards physiological signals, the autonomic nervous system (ANS) [20,21] signals have been increasingly emphasized, such as ECG, GSR, EMG, PPG, respiration (RSP), blood volume pulse (BVP), skin temperature (SKT), skin conductance (SC) and so on.

Picard (2001) collected four physiological signals by measuring EMG, BVP, GSR and RSP [7]. L Li (2006) proposed to recognize emotion using four physiological signals (ECG, SKT, SC and RSP) [8]. JS Tsai (2009) proposed an emotion recognition system with consideration of facial expression and physiological signals including SC, finger temperature and heart rate [9]. Previous studies have shown that emotion recognition through the ANS signals is feasible and effective.

In this paper, four physiological signals including ECG, GSR, facial EMG and PPG were collected. Here we briefly explain the underlying correlation between emotion and these physiological signals.

ECG and PPG are two of the most important physiological signals which have been subject to a high degree of attention in emotion recognition field. They are the digital representation of heart activity. The heart rate can reflect a person’s emotion state to a certain degree, for example, both surprise and stress induce physiological response: elevate heart rate substantially [22].

Facial EMG is a signal that can be easily measured from the face surface. It is caused by the activity of facial muscle contraction and reflects the state of the nerves muscle. It can reflect the changes of emotion from facial expressions.

GSR is another signal that can be measured from the body surface and reflects the electrical conductivity of the skin. The skin conductance level is closely related to emotion and attention [23,24,25]. The principle is that emotion changes can lead to the relaxation and contraction of blood vessels in the skin, as well as the secretion of sweat glands, thereby changing the conductivity of the skin.

### 2.2. Emotion Stimuli

The participants of our experiment are not professional actors, they are not good at expressing particular emotions. To obtain effective physiological signals, it is necessary to induce the targeted emotions of participants by emotion stimuli, as emotion stimuli has a great effect on the activity of the ANS [26].

At present, there are three widely used emotion induction techniques. One is to watch picture [27], video [28] or listen to music [29]. Another is to imitate reality, including sensory stimulation (such as darkness, noise stimulation), driving [30], human-machine interaction [31], playing computer games [32] and doing long and cumbersome cognitive tasks [10]. The other is to self-imagine and recall. It has been proved that these methods are effective to induce targeted emotions. Among them, the method using picture, audio and video is more common and convenient according to the investigation of related work. However, we conclude that visual stimulation using only picture and auditory stimulation using only music are not enough for effective emotion induction. Therefore, video clips were adopted as emotion stimuli in this paper, as video induction is a multimodal (audio, visual and cognitive) approach to evoke targeted emotions.

### 2.3. Mainstream Method

Considering the complexity of emotion-relevant features extracted from different physiological signals, many feature processing and classification methods were used to improve the performance of emotion recognition. Without dimension reduction, KH Kim (2004) directly adopted SVM as a pattern classifier to resolve emotion recognition of short-term physiological signals, correct classification ratios for 50 subjects were 78.4% and 61.8%, for the recognition of three and four categories, respectively [10]. J Cai (2009) used Tabu Search Algorithm (TS) for feature selection and proposed fisher-KNN for classification, the classification accuracy of affective (joy and sadness) ECG signal obtained from 391 subjects reached 90% [11]. B Cheng (2012) adopted SVM to serve as classifier, original physiological signals feature matrixes with the PCA dimension reduction approach were classified by the SVM, correct classification ratio of four emotion classes labeled by valence was 88.33%, the physiological dataset came from University of Augsburg, Germany [33] and included four physiological signals (EMG, SC and RSP) [12]. Yoon (2013) proposed the improved Bayes classifier using the weighted-log-posterior function to resolve the problem of the emotion recognition from electroencephalogram (EEG) signals, for the two-level class case, the average accuracies of the valence and arousal estimation were 70.9% and 70.1%, respectively [13].

These studies have made some achievements with the physiological dataset by using linear or partial nonlinear method. However, there are still some issues need to be resolved, such as the loss of feature information caused by feature reduction and low classification accuracy on multichannel physiological signals. To address these issues, a comprehensive nonlinear processing method was proposed in this paper.

## 3. Algorithm

**Figure 2:**
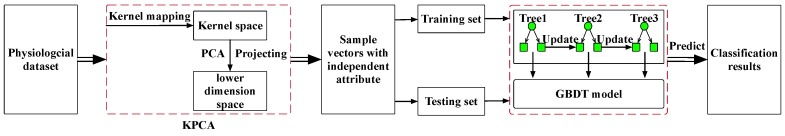
Block diagram of the comprehensive nonlinear processing method.

To improve the classification accuracy of the emotion recognition system, a comprehensive nonlinear processing method was proposed, which combined KPCA and GBDT for nonlinear feature processing and nonlinear classification, respectively. Figure 2 shows the procedure of the comprehensive nonlinear processing method. The input of the algorithm is the physiological dataset that containing 623 samples, each sample has 136 features. Firstly, KPCA is used for feature reduction. More concretely, the features of samples are mapped into a high-dimensional space called kernel space through the RBF kernel function. In the kernel space, it is easier to extract the nonlinear information of features and there may be some distinguishable features to be generated. After the kernel mapping, the standard PCA is performed, projecting the data from kernel space to a lower dimension space. At this point, we can get the sample vectors with independent attribute as input to GBDT model. Then, the sample vectors are divided into training set and test set according to 10-fold cross validation. The training set is used as input to update GBDT model, which is used as a nonlinear tree-based ensemble classifier to improve classification accuracy, since it can accomplish feature selection by choosing and combining significant features to generate a base learner (decision tree). At last, use the well trained GBDT model to predict the test set.

### 3.1. Kernel Principal Component Analysis

In the field of multivariate statistics, KPCA is a nonlinear extension of PCA using kernel methods. It is known for its good performance on a nonlinear dataset.

To understand the utility of KPCA [34,35,36], we suppose a dataset containing *N* data points, where $x_{i}\in R^{d}$. Unlike PCA, these data points are firstly mapped into a high-dimensional feature space F using a kernel function $k\left(x,y\right)$:

(1) $$x_{i}\overset{k\left(x,y\right)}{\to }\Phi \left(x_{i}\right).$$

The high-dimensional feature space is called kernel space. In the kernel space, to eliminate the influence of different feature scales, the data points are centered around the origin with

(2) $$\tilde{\Phi }\left(x_{i}\right)=\Phi \left(x_{i}\right)-\frac{1}{N}\begin{array}{c} \sum_{j=1}^{N}\Phi \left(x_{j}\right). \end{array}$$

Then standard PCA [37] is performed to project data into a lower dimension space through the projection vector α. In KPCA, any one eigenvector *V* of the covariance matrix is a linear combination of points $\tilde{\Phi }\left(x_{i}\right)$,

(3) $$V=\begin{array}{c} \sum_{k=1}^{N}\alpha_{i}\tilde{\Phi }\left(x_{i}\right) \end{array}$$

where $\alpha_{i}$ is the component of the projection vector α.

The commonly used kernel functions include linear kernel, polynomial kernel, and RBF (Gaussian) kernel. In this paper, the RBF kernel

(4) $$k\left(x,y\right)=e^{\frac{-{∥x-y∥}^{2}}{2\sigma^{2}}}$$

is adopted for its good performance on a nonlinear dataset.

Equation (1) shows that the number of nonlinear principal components extracted by KPCA can be substantially higher (up to the number of data points). This can be nearly always advantageous, especially in the situation where the dimension of the input data points is significantly smaller than the number of data points and a data structure is spread over all eigendirections [38].

**Figure 3:**
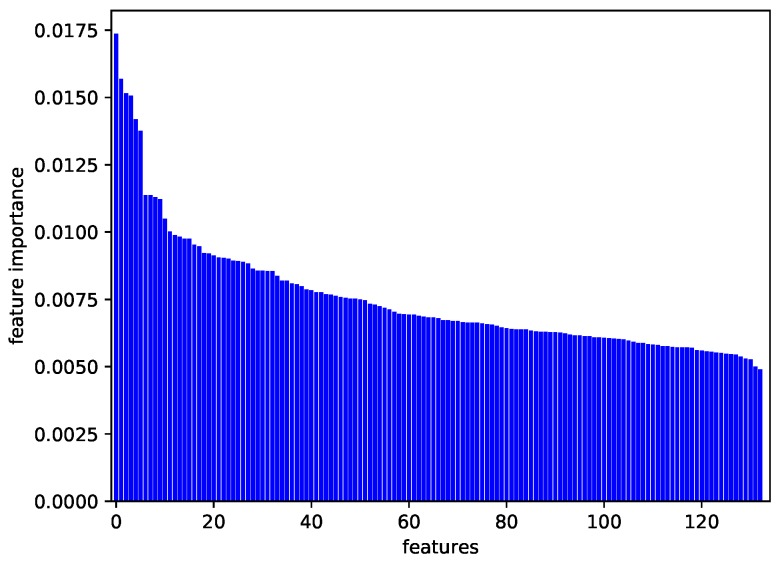
Feature importance.

**Figure 4:**
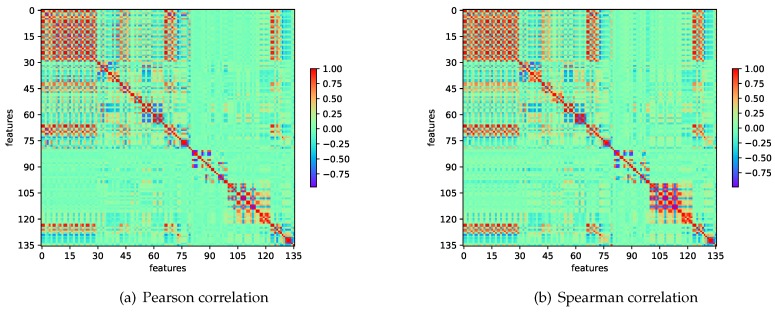
Pearson and Spearman correlations of features.

Our physiological dataset contains 623 samples and 136 dimensions described thoroughly in Section 4.3, just coincides this situation. Figure 3 shows the data structure that the importance of most features is similar, where the feature importance is evaluated by a tree-based ensemble method (ExtraTreesClassifier) [39]. The importance of each feature represents its contribution to the decision tree. The result on feature importance as depicted in Figure 3 shows that the contribution of each feature to the decision tree is not significantly different in classification. Under these circumstances traditional dimension reduction may lead to the loss of significant amounts of information [38]. That is one of the reasons for adopting KPCA. Another reason is that the emotion-relevant features are highly relevant with each other, the Pearson and Spearman correlations of features are shown in Figure 4. Pearson correlation coefficient (PCC) is a measure of the linear correlation between two variables *X* and *Y*. The formula is:

(5) $$\rho \left(X,Y\right)=\frac{cov\left(X,Y\right)}{\sigma_{X}\sigma_{Y}},$$

where $\rho \left(X,Y\right)$ is the PCC between *X* and *Y*, $\sigma_{X}$ is the standard deviation of *X* and $\sigma_{Y}$ is the standard deviation of *Y*. Spearman correlation coefficient is a nonparametric measure of rank correlation (statistical dependence between the rankings of two variables). The Spearman correlation is defined as the Pearson correlation between the rank variables [40]. These two correlation coefficients have a value between +1 and −1, where +1 is total positive linear correlation, 0 is no linear correlation, and −1 is total negative linear correlation.

Specifically, 136 features are mapped into infinite dimension space by RBF kernel mapping, to make the dataset easier to separate. Then standard PCA is performed to reduce feature dimension to 623 (the number of samples), as excessive dimension reduction may cause the loss of feature information.

### 3.2. Gradient Boosting Decision Tree

GBDT is an ensemble classifier constructed by multiple decision trees using boosting framework. It is a linear combination of the basic models (decision tree). The basic models are established by significant features and updated in the gradient descent direction of the pseudo-residuals. It has shown very good performance on the classification and regression tasks in many data mining competitions organized by KDD Cup and Kaggle. Considering its advantages of feature selection and predictive power [41,42,43], we can infer that GBDT will greatly improve the accuracy of emotion recognition on the nonlinear physiological dataset with large number of features.

To understand GBDT [41,44,45], we assume a training set $S={\left\{\left(x_{i},y_{i}\right)\right\}}_{i=1}^{n}$, a predict model $F\left(x\right)$, a differentiable loss function $L\left(y,F\left(x\right)\right)$. Firstly, the predict model is initialized with a constant value:

(6) $$F_{0}\left(x\right)=\arg\min_{\gamma }\begin{array}{c} \sum_{i=1}^{n}L\left(y_{i},\gamma \right) \end{array}.$$

Then compute pseudo-residuals:

(7) $$r_{im}=-{\left[\frac{\partial \left(y_{i},F\left(x_{i}\right)\right)}{\partial F\left(x_{i}\right)}\right]}_{F\left(x\right)=F_{m-1}\left(x\right)}i=1,\cdots n$$

where *m* represents the number of iteration. Fit a base learner (decision tree) $h_{m}\left(x\right)$ in the gradient descent direction of the pseudo-residuals. Next the minimum value of the loss function is searched linearly with:

(8) $$\gamma_{m}=\arg\min_{\gamma }\begin{array}{c} \sum_{i=1}^{n}L\left(y_{i},F_{m-1}\left(x_{i}\right)+\gamma h_{m}\left(x_{i}\right)\right) \end{array}.$$

At last, update the model with $h_{m}\left(x\right)$:

(9) $$F_{m}\left(x\right)=F_{m-1}\left(x\right)+\nu \gamma_{m}h_{m}\left(x\right),0<\nu <1$$

where *v* is the learning rate.

## 4. Experiment

### 4.1. Collection of Multiple Physiological Signals

To acquire a high-quality database of physiological signals, a scientific and reasonable arrangement was made for the experiment from the selection of participants, the settings of experiment instruments, the selection of emotion induced videos and the construction of experiment scene.

#### 4.1.1. Participants and Emotion Stimuli

The participants include 29 students, 15 males and 14 females, which come from software college, Tianjin University, China. They are aged from 18 to 30 years (mean = 22.97, standard deviation = 2.83). Health survey questionnaire shows that they have no history of medical, neurological, or psychiatric illness. They are so healthy both physically and psychologically that they can express emotions normally. The experiment was carried out on the premise that the participants were informed of the purpose and details of the experiments. In addition, consent forms signed by all participants have been obtained before the experiment.

For the stimuli, we choose a movie clip of Diors Man for pleasure, a movie clip of Grudge for fear, a movie clip of the Aftershock for sadness and a movie clip of Silenced for anger. These representative movie clips are selected from 20 movie clips by online voting of 50 non-subjects. Scientific research indicates that a person’s concentration of energy lasts about 20 min [46]. Considering the participants’ patience, each movie clip plays about 4 min in pleasure, fear, sadness, and anger order. In addition, the internal of each two clips plays a one-minute landscape pictures to make the participants calm down, the time and pictures are voted by participants. The whole process of stimuli lasts about 20 min.

#### 4.1.2. Experiment Instruments and Scene

The experiment was arranged in a closed and quiet room. The physiological signals including ECG, GSR, EMG and PPG were acquired using BIOPAC MP150 system.

MP150 multichannel physiological recorder of American BIOPAC company is the most widely used physiological record analysis system in the world. A MP150 system consists of a host computer, acquisition and analysis software, and various amplifiers, sensors, leads and electrodes. The hardware of MP150 system is modular, which can measure different physiological signals at random. The system can also capture video synchronously and synchronize with the third-party equipment such as the eye tracker. The supporting software AcqKnowledge can view, measure, analyze and transform data in real time. It can also set different sampling rates and output multiple file formats, including AcqKnowledge graph, Excel, MATLAB, Text, etc.

The sampling rate was fixed at 200 Hz for all channels of all signals. Each signal has the corresponding sensor and amplifier. Amplifier settings include high-pass filter, low-pass filter, and gain.

ECG collection used 2 screened leads (LEAD110S), 1 unscreened lead (LEAD100), 3 disposable electrodes and ECG100C amplifier. The shields of the 2 screened leads were connected to the shield of the amplifier. Positive electrode (VIN+) was connected to the left lower extremity, negative electrode (VIN-) was connected to the right upper extremity and the ground electrode (GND) was connected the right lower extremity. Amplifier gain was set at 500, high-pass filter was set at 0.5 Hz and low-pass filter was set at 35Hz ON.

GSR collection used Skin Resistance Sensor (TSD203) and GSR100C amplifier. TSD203 consisted of two non-polarized electrodes, which were attached to the fingertips through the bandage. Amplifier gain was set at 1000, high-pass filter was set at DC (Direct Current) and low-pass filter was set at 1Hz.

EMG collection used 2 LEAD110S, 1 LEAD100, 3 disposable electrodes and EMG100C amplifier. VIN+ and VIN- were connected to the forehead, GND was connected to the back of the ear. Amplifier gain was set at 2000, high-pass filter was set at 1Hz and low-pass filter was set at 100Hz HPN OFF.

PPG collection used PPG Pulse Sensor (TSD200) and PPG100C amplifier. The TSD200 was also connected to the fingertip. Amplifier gain was set at 500, high-pass filter was set at 0.05 Hz and low-pass filter was set at 10 Hz.

**Figure 5:**
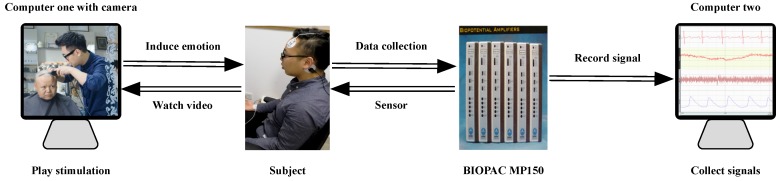
The diagram of experiment platform.

Besides, we used two computers. One computer was used to record signals from BIOPAC MP150 system, another computer with a camera was used to play emotion stimuli and record the facial expressions of the participants synchronously. Figure 5 shows the experiment platform.

**Figure 6:**
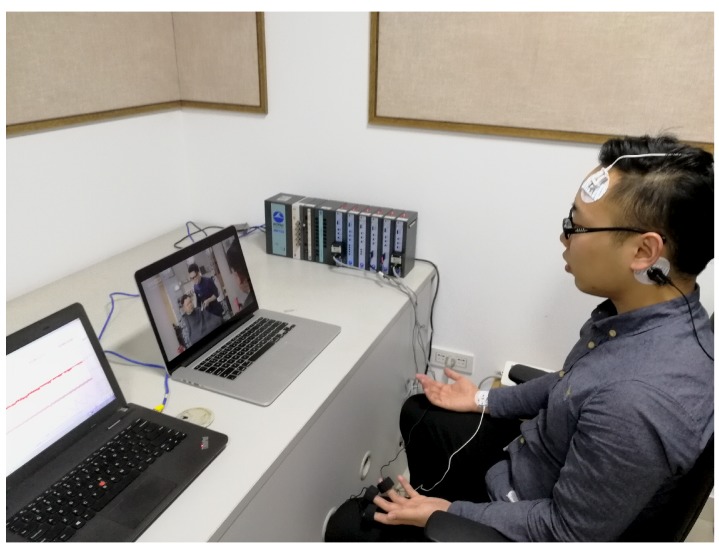
A participant in emotion induction experiment.

At the beginning of experiment, we explained the experiment process for participants and helped them wear physiological signal sensors. Subsequently, about 20 min video was played. As the experiment began, the screen of computer used for recording physiological signals was blocked to avoid interference to the subjects. The participants were requested to be as relaxed as possible during this period. Figure 6 shows a participant in the emotion induction experiment. After the experiment, participants were asked to fill out the feedback form about emotion experience. In the subsequent steps, the emotion reports and the facial expressions of the participants will be used for the emotion label of physiological signals.

### 4.2. Preprocessing

Physiological signals are easily interfered by noise, the electromagnetic phenomenon of the experiment instrument, the power frequency, and the action of participants. Therefore, the preprocessing to physiological signals is an indispensable step in emotion recognition.

Firstly, the N-tap FIR (finite impulse response) adaptive filter with coefficients updated using least means squares feedback was used to eliminate interference between different channels, the order and step size of the filter were 5 and 1 × 10${}^{-6}$, respectively. Then to reduce the required length of signals, we intercepted about 20s signal segments of each sample according to the participants’ emotion report and facial expression. The subsequent processing was all based on these signal segments.

**Figure 7:**
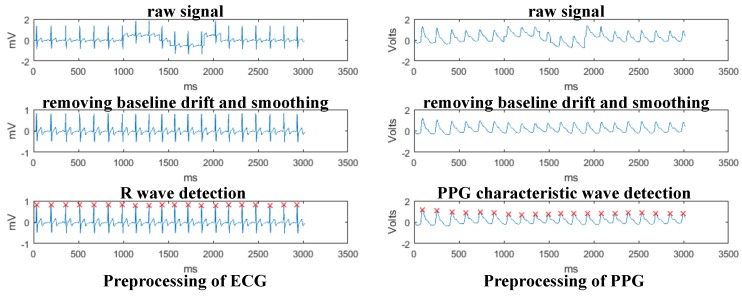
The preprocessing of ECG and PPG signals.

For ECG and PPG signals, baseline drift is a serious interference, which is often caused by limb movement, respiratory movement, poor electrode contact and so on. Considering this problem, the moving average method was used for smoothing, wavelet transform was used to remove baseline drift and detect characteristic waves (R wave in ECG and PPG characteristic wave). Specially, the original ECG signal was firstly decomposed into 7 layers using db5 wavelet basis, and the approximate signal was obtained as close as possible to the ECG baseline drift noise. Then, the approximate signal was averaged and reconstructed from the detail signal to eliminate the interference caused by the baseline drift. After that, the wavelet transform of ECG signal on scale 8 produced a pair of modulus maxima. The peak time of R wave corresponds to the zero-intersection point of the modulus maxima pair, since the energy of R wave is mainly concentrated on scale 8. At this point, we got the characteristic wave of ECG after eliminating baseline drift. The processing of PPG is the same as ECG signal, except that use sym8 wavelet basis to eliminate baseline drift by 5 layers decomposition and detect the PPG characteristic wave on scale 4. According to the above method, we processed a subject’s ECG and PPG signals, which were interfered by baseline drift due to the body movement of the subject. Figure 7 shows the result of the process to ECG and PPG signal. For EMG signal, a Butterworth low-pass filter with 0.4 Hz was used to denoise. For GSR signal, a Butterworth low-pass filter with 0.3 Hz was used for smoothing.

### 4.3. Feature Extraction

Augsburg Biosignal Toolbox (AuBT) [33] is a toolbox for analyzing physiological signals in the face of emotion recognition. After preprocessing, emotion-relevant features were extracted from physiological signal segments using AuBT.

A typical ECG signal is composed of P wave, Q wave, R wave, S wave and T wave. The features of ECG include the time interval and amplitude characteristic of each wave, heart rate and heart rate variability (HRV). HRV is the variation in the time interval between heartbeats. Usually, the RR interval time series is used as the equivalent of the HRV time series [47]. Amplitude characteristics refer to the statistical features of P, R and S amplitude. Heart rate is generally used to distinguish positive and negative emotions. HRV refers to the concussion of the time interval of a continuous heartbeat, which reflects the psychological stress of adults. The initial features include 13 underlying characteristics (intervals of each characteristic wave between the adjacent heartbeat, amplitude, HRV) and the high-level features extracted from the 13 underlying features. Table 1 presents the details of ECG features. pNN50 is a common indicator of HRV, which represents number of pairs of adjacent RR intervals differing by more than 50 ms divided by the total number of RR intervals. Triind is the total number of all RR intervals divided by the height of the histogram of all RR intervals measured on a discrete scale with bins of 7.8125 ms.

21 time domain and frequency domain features are extracted from GSR signal, including three underlying features: the raw signal GSR, the first order differential and the second order differential of GSR. Each underlying feature consists of 7 statistical features, namely, mean, median, std, min, max, minRatio and maxRatio. Table 2 presents the details of GSR features.

The feature extraction of EMG signal is the same as GSR signal. Table 3 presents the details of EMG features.

PPG features include the statistical features of its characteristic wave amplitude and the pulse rate variability (PRV) features. PRV is the variation in the time interval between pulse beats. Table 4 presents the details of PPG features.

After the feature extraction, some features have almost the same values in all samples, such as the mean value of GSR-1Diff and GSR-2Diff. These features are unhelpful to emotion recognition from signals. Removing these features with variance less than 0.8 × (1 − 0.8), there are total 136 features, including 80 ECG features, 20 EMG features, 17 GSR features and 19 PPG features. As can be seen from the feature extraction described above, some features from the same signal are strongly related. Some researchers have applied feature selection algorithms to filter out these redundant and strongly related features [11,48], but this paper did not do this, as KPCA can remove the correlation between features automatically, which was one of the reasons why we adopted KPCA for feature reduction. These features from multichannel physiological signals are complex and interrelated, making the physiological dataset difficult to separate. The emotion reports and facial expressions of participants were used for emotion annotation. Then, the physiological dataset was constructed by these features and emotion labels. As the participants have different sensitivity and response to each emotion, our physiological dataset is a little unbalanced. The number of samples on pleasure, fear, sadness, and anger are 105, 185, 168, and 165, respectively.

### 4.4. Results Analysis

In this section, a lot of contrast experiments were conducted to verify the effectiveness of the comprehensive nonlinear method by 10-fold cross validation. Effectiveness was measured by classification accuracy, that was the ratio of samples correctly classified in the test set. For contrast, we employed two traditional feature reduction methods (PCA and Locally Linear Embedding (LLE)) and three frequently used classifiers (SVM, KNN and Gaussian Naive Bayes (GaussianNB)).

Table 5 shows the classification accuracy of different models on the features of each signal. From the last line of the table, we can easily find that all the classifiers based on traditional dimension reduction methods have a poor performance on our physiological dataset. However, the use of KPCA greatly improves their classification performance. Therefore, we infer that KPCA has great advantages in handling nonlinear multichannel physiological features. Since our physiological dataset is composed of features from different signals, the interaction and nonlinear relation between different features in classification must be considered. GBDT can interact with multiple sets of features automatically and has good nonlinear classification ability, so it should have a good performance on the multichannel physiological dataset. Classification results show GBDT outperforms other classifiers (SVM, KNN and GaussianNB) in all situations, demonstrating its predictive power on the multichannel physiological dataset and proving our analysis.

Besides, comparing the classification accuracy of different signals in Table 5, it can be found that ECG has the highest classification accuracy in most models. Therefore, we consider that ECG has rich emotion-relevant features, which can clearly reflect changes in human emotions. Many researchers have used ECG for emotion recognition [11,49,50]. We can also see that the classification performance of the comprehensive nonlinear processing model (KPCA & GBDT) on multichannel physiological signals is better than that on single signal. Therefore, we think the comprehensive nonlinear processing model can improve the accuracy of emotion recognition towards complex physiological datasets (nonlinear and multichannel).

Table 6 shows the classification accuracy of different models on each emotion. The classification accuracy of each emotion represents the ratio at which the samples belonging to this emotion are correctly classified. Obviously, in most models, fear has the highest classification accuracy in four emotions. Therefore, it can be considered that the physiological responses of the participants are more intense when they are scared, or the fear emotion is more easily evoked than pleasure, sadness, and anger.

To get the final hyper parameters of GBDT, we tuned the boosting and tree-specific parameters of GBDT. learning_rate and n_estimators are boosting parameters. learning_rate is the weight reduction coefficient of each decision tree, which controls the amplitude of variation of the decision tree estimation, the lower the value is, the better the generalization is. n_estimators is the number of decision tree and the number of boosting stages to perform. Generally, increasing n_estimators can improve the performance and robustness of the model without overfitting. It is often tuned together with learning_rate, lower learning_rate usually requires more n_estimators. max_depth and max_features are tree-specific parameters, correspond to the maximum depth and the largest number of features of each decision tree. Increasing max_depth and max_features appropriately can also improve the performance of the model, because there are more features to be considered at each node of the decision tree. However, this is not entirely correct, because it reduces the diversity of individual decision trees. Therefore, the values of max_depth and max_features should be adjusted according to the distribution of the dataset. The best GBDT model achieves 93.42% accuracy with learning_rate=0.1, n_estimators=200, max_depth=4 and max_features=30. Table 7 gives a summary to the performance of different hyper parameters with KPCA & GBDT. As is evident from the table, learning_rate, n_estimators, max_depth and max_features all influence the classification accuracy. Since the physiological dataset has many features (623) after KPCA processing, it is necessary to enlarge the size of decision tree to improve the recognition accuracy. From the table, it can be seen that increasing the values of max_depth and max_features can improve the recognition accuracy, that is consistent with the characteristics of the physiological dataset and above analysis. Besides, under the condition that the parameters of decision tree are fixed, the recognition accuracy can also be improved by increasing the step size of decision tree fitting and the number of iterations appropriately without overfitting, as shown in the result of learning_rate and n_estimators in Table 7.

At last, GaussianNB and GBDT were adopted to verify the influence of the number of nonlinear principal components (n_components) on classification. The results are shown in Table 8. As the original dimension of our physiological dataset is 136, GaussianNB reaches the highest classification accuracy on 300 principal components and GBDT reaches the highest classification accuracy on 600 principal components. We consider that the large number of nonlinear principal components generate more distinguishable features and reduce the loss of feature information, and it is not achievable by traditional dimension reduction method. Besides, the table also shows that in the higher dimension space (n_components>300), the accuracy of GaussianNB model will degrade, but the accuracy of GBDT still improves gradually, it is a good illustration for the superiority of GBDT in processing high-dimensional data. For the degradation of GaussianNB performance, it can be inferred that there may be some interference features in classification with the increase of principal components. For the improvement of GBDT performance, considering the construction of GBDT model, we think this is because there are more features to be considered at each node of the decision tree with the increase of principal components, which is similar to the principle of parameters max_depth and max_features. This also proves the power of feature selection of GBDT.

To validate the performance of the comprehensive nonlinear processing method on classification of the physiological dataset, different experiments based on KPCA and GBDT were conducted. Results in Table 5, Table 6 and Table 8 illustrate the superiority of the comprehensive nonlinear method on emotion recognition towards the nonlinear multichannel physiological dataset.

## 5. Conclusions

This paper proposed a comprehensive nonlinear processing method to overcome the difficulty of emotion recognition towards a nonlinear multichannel physiological dataset. Firstly, KPCA was adopted to map the data into a high-dimensional space and then reduce dimension. Next, GBDT was used to recognize emotions from a nonlinear multichannel physiological dataset with many features. To investigate the performance of the method, experiments were carried out on four physiological signals, which were collected from 29 participants under four induced emotions. The comprehensive nonlinear processing method outperformed general models on a nonlinear multichannel physiological dataset. The classification accuracy was 93.42% on the four categories. As physiological signals are complex, more effective emotion-relevant features should be extracted from multiple physiological signals in the future emotion recognition system.

## Figures and Tables

**Table 1:** Features extracted from ECG signal.

Underlying Features	Statistical Features
RR interval	Mean, Median, Std, Min, Max, Range
PP interval	Mean, Median, Std, Min, Max, Range
QQ interval	Mean, Median, Std, Min, Max, Range
SS interval	Mean, Median, Std, Min, Max, Range
TT interval	Mean, Median, Std, Min, Max, Range
PQ interval	Mean, Median, Std, Min, Max, Range
QS interval	Mean, Median, Std, Min, Max, Range
ST interval	Mean, Median, Std, Min, Max, Range
P amplitude	Mean, Median, Std, Min, Max, Range
R amplitude	Mean, Median, Std, Min, Max, Range
S amplitude	Mean, Median, Std, Min, Max, Range
HRV	Mean, Median, Std, Min, Max, Range, pNN50, the mean of frequency spectrum
HRV distribution	Mean, Median, Std, Min, Max, Range, Triind

**Table 2:** Features extracted from GSR signal.

Underlying Features	Statistical Features
Raw GSR	Mean, Median, Std, Min, Max, MinRatio, MaxRatio
GSR-1Diff	Mean, Median, Std, Min, Max, MinRatio, MaxRatio
GSR-2Diff	Mean, Median, Std, Min, Max, MinRatio, MaxRatio

**Table 3:** Features extracted from EMG signal.

Underlying Features	Statistical Features
Raw EMG	Mean, Median, Std, Min, Max, MinRatio, MaxRatio
EMG-1Diff	Mean, Median, Std, Min, Max, MinRatio, MaxRatio
EMG-2Diff	Mean, Median, Std, Min, Max, MinRatio, MaxRatio

**Table 4:** Features extracted from PPG signal.

Underlying Features	Statistical Features
P-PPG	Mean, Median, Std, Min, Max, Range
PRV-PPG	Mean, Median, Std, Min, Max, Range, Frequency spectrum

**Table 5:** Classification results of different models on the features of each signal.

Accuracy (%)	SVM			KNN			GaussianNB			GBDT		
	LLE	PCA	KPCA	LLE	PCA	KPCA	LLE	PCA	KPCA	LLE	PCA	KPCA
ECG(80)	**43.49**	35.23	**67.43**	**40.94**	**42.70**	41.56	**35.15**	34.64	**73.03**	**42.05**	**47.66**	**83.14**
EMG(20)	29.86	31.16	33.72	26.49	33.87	33.23	34.19	32.43	36.78	30.48	38.21	33.70
GSR(17)	38.05	**38.69**	35.48	35.95	33.55	**45.60**	33.87	**40.27**	32.73	35.30	43.67	35.46
PPG(19)	34.21	31.30	32.75	33.39	34.34	34.37	31.62	37.88	26.00	35.50	40.44	36.44
Total(136)	43.02	39.22	**61.49**	43.80	43.01	**63.70**	38.53	39.01	**88.13**	44.46	54.73	**93.42**

**Table 6:** Classification results of different emotions.

Accuracy (%)	SVM			KNN			GaussianNB			GBDT		
	LLE	PCA	KPCA	LLE	PCA	KPCA	LLE	PCA	KPCA	LLE	PCA	KPCA
Pleasure	33.01	30.04	63.02	21.63	26.38	66.07	28.76	34.92	92.44	31.43	46.31	89.91
Fear	**54.64**	**80.27**	67.68	**62.38**	**62.73**	**83.91**	45.71	22.94	**92.73**	**93.80**	**65.78**	96.71
Sadness	44.71	22.33	53.16	38.76	42.57	51.40	**55.87**	**78.07**	77.24	45.38	51.54	92.41
Anger	36.14	21.64	**69.77**	44.12	33.98	46.68	21.90	15.70	92.34	39.42	49.28	**98.29**

**Table 7:** The detailed classification results of KPCA & GBDT.

learning_rate	n_estimators	max_depth	max_features	Accuracy (%)
0.1	50	2	10	86.05
0.1	50	3	10	88.75
0.1	50	4	10	89.25
0.1	50	4	20	90.69
0.1	50	4	30	91.50
0.1	100	4	30	92.46
0.1	200	4	30	**93.42**
0.01	200	4	30	92.46
0.001	200	4	30	89.57

**Table 8:** The classification results based on the different number of principal components.

n_components	GaussianNB Accuracy (%)	GBDT Accuracy (%)
10	51.34	49.75
30	64.23	77.52
50	78.81	88.75
100	78.81	86.99
200	82.34	84.43
300	**88.13**	86.51
400	86.67	86.34
500	87.96	92.14
600	88.12	**93.10**
